# Biophysical Characterization of Epigallocatechin-3-Gallate Effect on the Cardiac Sodium Channel Na_v_1.5

**DOI:** 10.3390/molecules25040902

**Published:** 2020-02-18

**Authors:** Mohamed-Yassine Amarouch, Han Kurt, Lucie Delemotte, Hugues Abriel

**Affiliations:** 1 R.N.E Laboratory, Multidisciplinary Faculty of Taza, University Sidi Mohamed Ben Abdellah of Fez, Fez 30000, Morocco; 2 Science for Life Laboratory, Department of Applied Physics, KTH Royal Institute of Technology, SE-100 44 Solna, Sweden; han.kurt@outlook.com (H.K.); lucie.delemotte@scilifelab.se (L.D.); 3 Institute of Biochemistry and Molecular Medicine (IBMM), University of Bern, 3012 Bern, Switzerland

**Keywords:** EGCG, Na_v_1.5, cellular electrophysiology, molecular dynamics, ion channels

## Abstract

Epigallocatechin-3-Gallate (EGCG) has been extensively studied for its protective effect against cardiovascular disorders. This effect has been attributed to its action on multiple molecular pathways and transmembrane proteins, including the cardiac Na_v_1.5 channels, which are inhibited in a dose-dependent manner. However, the molecular mechanism underlying this effect remains to be unveiled. To this aim, we have characterized the EGCG effect on Na_v_1.5 using electrophysiology and molecular dynamics (MD) simulations. EGCG superfusion induced a dose-dependent inhibition of Na_v_1.5 expressed in tsA201 cells, negatively shifted the steady-state inactivation curve, slowed the inactivation kinetics, and delayed the recovery from fast inactivation. However, EGCG had no effect on the voltage-dependence of activation and showed little use-dependent block on Na_v_1.5_._ Finally, MD simulations suggested that EGCG does not preferentially stay in the center of the bilayer, but that it spontaneously relocates to the membrane headgroup region. Moreover, no sign of spontaneous crossing from one leaflet to the other was observed, indicating a relatively large free energy barrier associated with EGCG transport across the membrane. These results indicate that EGCG may exert its biophysical effect via access to its binding site through the cell membrane or via a bilayer-mediated mechanism.

## 1. Introduction

Ion channels are pore-forming transmembrane proteins involved with the transport of ions through cell membranes. Their activity is essential for the excitation-contraction coupling in cardiac cells. In this sense, the voltage-gated sodium channels generate the rapid action potential upstroke of cardiomyocytes and thereby play a crucial role in heart excitability and conduction.

Within the nine isoforms of voltage-gated sodium channels, Na_v_1.5 displays a preponderant expression in cardiac cells [1]. This isoform is composed of intracellular N and C terminal tails and four homologous domains (DI-DIV), each consists of six transmembrane segments (S1–S6). These domains fold together with their S5-S6 segments to build a highly selective Na^+^ pore [2,3].

Na_v_1.5 dysfunction is linked with an increasingly wide range of inherited cardiac arrhythmias [4,5]. Gain-of-function mutations of this channel are associated with many disorders such as the congenital long QT syndrome, multifocal ectopic Purkinje-related premature contractions (MEPPC), and exercise-induced polymorphic ventricular tachycardia [6,7,8,9,10,11]. Whereas the loss-of-function mutations are linked with Brugada syndrome, sick sinus syndrome, and cardiac conduction disease [4,5,12]. In the same line, mutations in genes encoding proteins that regulate, directly or indirectly, Na_v_1.5 function are associated with many cardiac arrhythmias [13]. This is true for many Na_v_1.5-interacting proteins such as Na_v_-β subunits, Plakophillin-2, and more recently for the nuclear proteins Lamin A/C [14,15,16,17]. As such, Na_v_1.5 channels become highly targeted by several drug classes, including anti-arrhythmic drugs [18], despite their associated side effects [19].

In this context, bioactive molecules extracted from medicinal plants are pledged to offer added values in preventing arrhythmic episodes. Epigallocatechin-3-Gallate (EGCG), the predominant polyphenol of green tea, was shown to be a promising natural alternative in the setting of cardiac arrhythmia [20,21].

Green tea extracts and EGCG have been extensively studied for their protective effects against cardiovascular disorders. Many authors have shown that green tea consumption is associated with a lower risk of heart disease and reduced cardiovascular mortality [22,23]. Moreover, several experimental studies revealed that EGCG reduces chronic ventricular remodeling after myocardial ischemia in rats [24], improves left ventricular dysfunction, suppresses myocardial inflammation in rat autoimmune myocarditis [25], modulates arrhythmogenic activity, and calcium homeostasis of rabbit left atrium [21], and inhibits angiotensin II-induced cardiomyocyte hypertrophy [26].

The cardiac activity of EGCG has been attributed to its effect on multiple intracellular molecular pathways and transmembrane proteins [20,21,22,26,27,28], including Na_v_1.5. This compound has shown a dose-dependent inhibitory effect on the cardiac sodium current I_Na_ [21,29,30]. However, the molecular and biophysical mechanism underlying this effect remains to be unveiled. To this aim, we have worked out to characterize the mechanisms underlying the EGCG effect on the cardiac sodium channel Na_v_1.5.

## 2. Results

### 2.1. Inhibitory Effect of EGCG on Human Na_v_1.5 Channels

**Figure 1:**
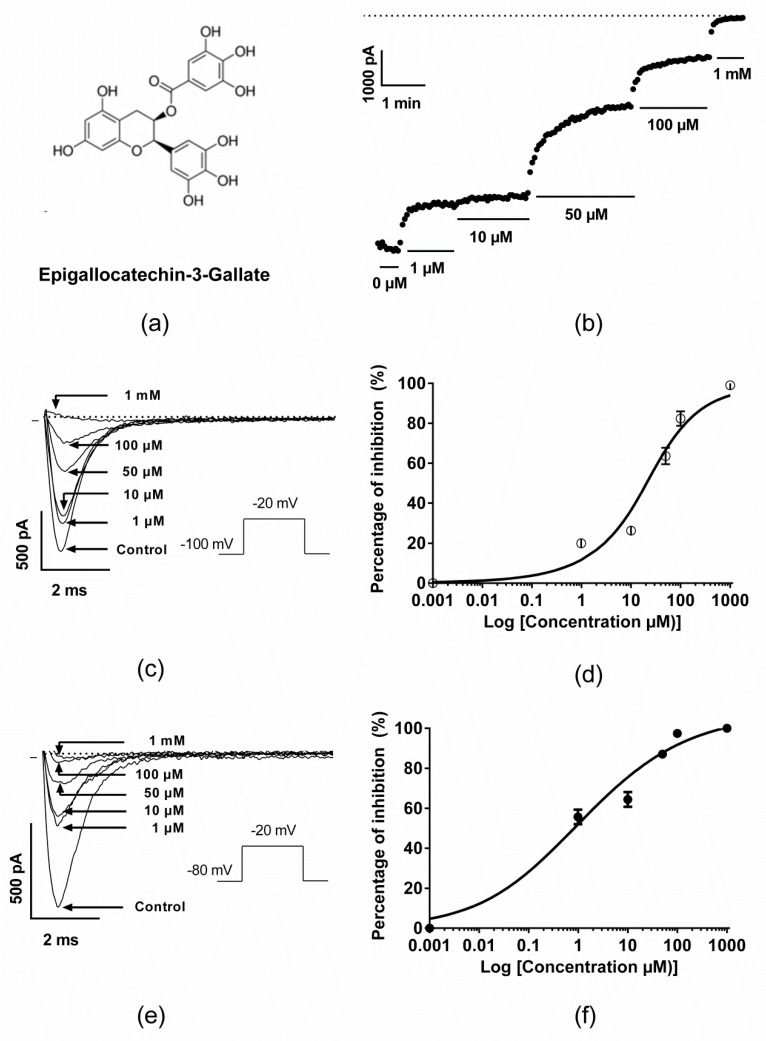
Pharmacological effects of Epigallocatechin-3-Gallate (EGCG) on the cardiac sodium channel Na_v_1.5. (**a**) Chemical structure of EGCG. (**b**) Time course recording of the I_Na_ current in the presence of various concentrations of EGCG. The dashed line represents the zero current. (**c**) Representative traces of I_Na_ currents in the presence of various EGCG concentrations at holding potential −100 mV. (**d**) Dose-response effects of EGCG on the inhibition of I_Na_ peak currents (holding potential = −100 mV, IC_50_ = 31.2 ± 3.6 µM, *n* = 8). (**e**) Representative traces of I_Na_ currents in the presence of various EGCG concentrations at holding potential −80 mV. (**f**) Dose-response effects of EGCG on the inhibition of I_Na_ peak currents (holding potential = −80 mV, IC_50_ = 2,1 ± 1 µM, *n* = 5–7).

The pharmacological effects of EGCG (Figure 1a) on the cardiac sodium channel isoform were investigated using tsA201 cells transiently transfected with the human Na_v_1.5 channel in the presence of its regulatory subunit Na_v_-β1. The latter is known for its effect on increasing the functional expression of Na_v_1.5 channels in many heterologous systems [14]. I_Na_ currents were generated by 15 ms step depolarization to −20 mV from a holding potential of −100 or −80 mV at a frequency of 0.33 Hz. As previously reported by Kang et al. [30], EGCG superfusion induced a dose-dependent inhibition of the Na_v_1.5 channel. The IC_50_ for the EGCG inhibition was 31.2 ± 3.6 µM when measured from the −100 mV holding potential (Figure 1b–d) and 2.1 ± 1 µM when measured at −80 mV (Figure 1e,f). On the other hand, EGCG dose-dependent effect on the fast inactivation kinetics was investigated. This resulted in slowing the inactivation kinetics as long as the EGCG concentrations increased, mainly at 50 and 100 µM (Supplementary Figure S1).

### 2.2. Effect of EGCG on the Voltage-Dependence Properties of Na_v_1.5 Channels

**Figure 2:**
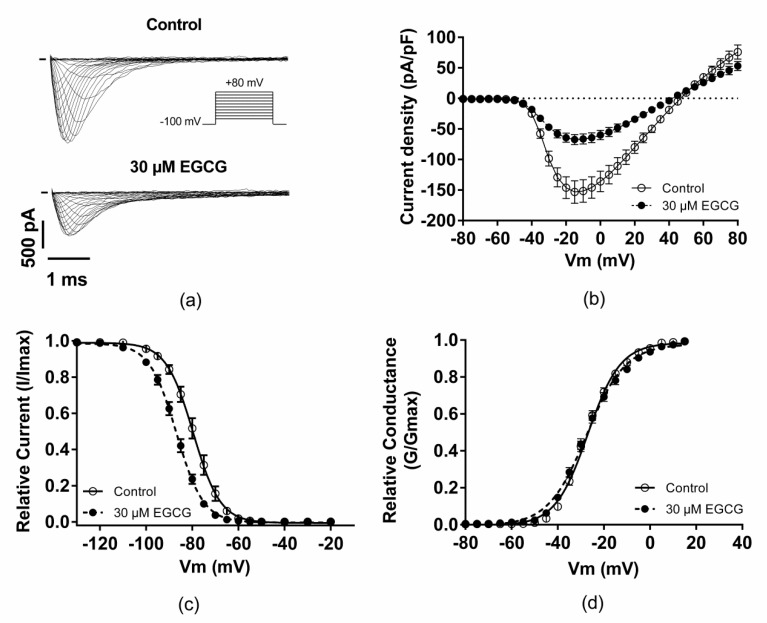
Effect of EGCG on the gating properties of Na_v_1.5 channels. (**a**) Representative traces of I_Na_ current in the presence or the absence of 30 µM of EGCG. (**b**) I/V relationship in the presence or the absence of EGCG. (**c**,**d**) Effect of EGCG on the voltage-dependence of inactivation and activation, respectively.

**Figure 3:**
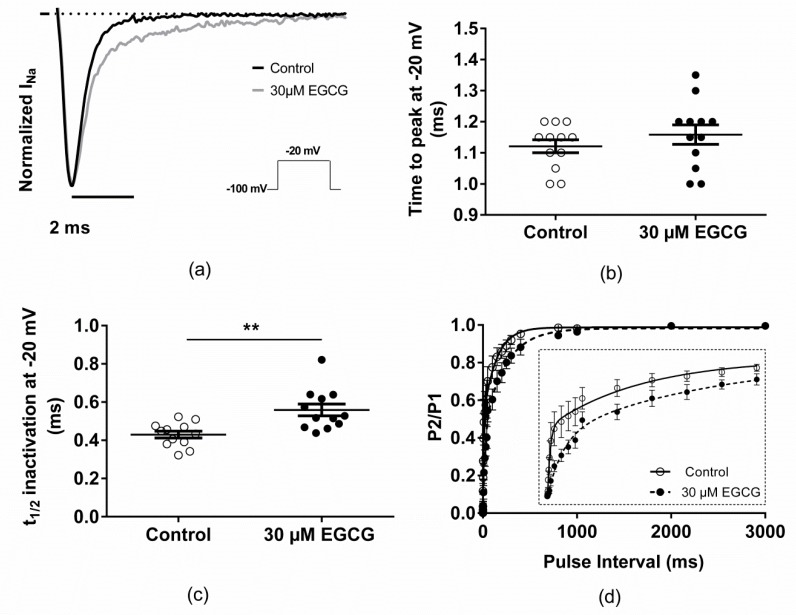
Effect of EGCG on I_Na_ kinetics. (**a**) Representative traces of normalized I_Na_ current in the presence or the absence of 30 µM of EGCG. I_Na_ currents were normalized to the maximal peak current measured, in each condition, at −20 mV. EGCG effect on I_Na_ time to peak (**b**), inactivation kinetics (**c**), and recovery from fast inactivation (**d**; inset, zoom on the interval between 0 and 300 ms).

The second step of EGCG pharmacological characterization was to study the compound effect on Na_v_1.5 biophysics. For this aim, the voltage-dependence properties of Na_v_1.5 channels were investigated in the presence or absence of 30 µM EGCG. The superfusion of this compound inhibited the cardiac sodium channels (Figure 2a,b, Table 1), induced a shift of steady-state inactivation towards more negative potentials (Figure 2c, Table 1), slowed the inactivation kinetics (Figure 3a,c, Table 1), and delayed the recovery from fast inactivation (Figure 3d, Table 1). Moreover, EGCG did not modify the voltage-dependence of activation but significantly affected the activation curve slope (Figure 2d, Table 1).

### 2.3. EGCG did not Produce any Use-Dependent Blockade of Na_v_1.5 Channels

To characterize the potential use-dependent block (UDB) of EGCG on I_Na_ current, the effect of rapid pulsing on Na_v_1.5 was investigated via the application of a series of 50 15 ms depolarizing pulses from −100 to −20 mV at various stimulation rates (1 and 10 Hz). After the establishment of the whole-cell configuration, cells were allowed to stabilize before the first run of the UDB protocol in the absence of EGCG. Then, EGCG was perfused for 3 min while the membrane potential was held at −100 mV, followed by the UDB protocol under 30 µM EGCG.

**Figure 4:**
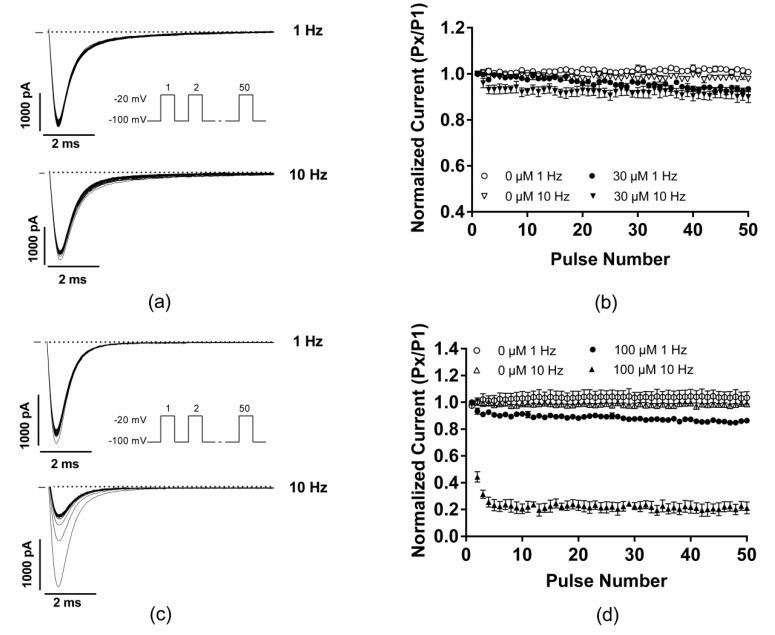
Use-dependent block protocol in the presence or absence of 30 µM EGCG. (**a**) Representative trace of I_Na_ currents at 1 and 10 Hz in the presence of 30 µM of EGCG. (**b**) Normalized I_Na_ current versus pulse number in the presence or absence of 30 µM of EGCG at 1 and 10 Hz. (**c**) Representative trace of I_Na_ currents at 1 and 10 Hz in the presence of 100 µM of Mexiletine. (**d**) Normalized I_Na_ current versus pulse number in the presence or absence of 100 µM of mexiletine at 1 and 10 Hz.

After the application of EGCG, I_Na_ currents were slightly but significantly reduced by 7% and 8% compared with the control when pulsing at 1 and 10 Hz, respectively (Figure 4a,b, Table 2). These results suggest that there is little or no use-dependent blockade of EGCG on Na_v_1.5 related currents.

To validate our experimental conditions, especially the used UDB protocol, mexiletine has been used as a positive control. As shown in Figure 4c,d, the application of 100 µM mexiletine significantly reduces the I_Na_ current by 17% and 77% compared with the control when pulsing at 1 and 10 Hz, respectively. These findings demonstrated the ability of the applied UDB protocol to characterize the use-dependent block of a given drug.

### 2.4. MD Simulations Suggest that EGCG does not Spontaneously Permeate Cell Membranes

**Figure 5:**
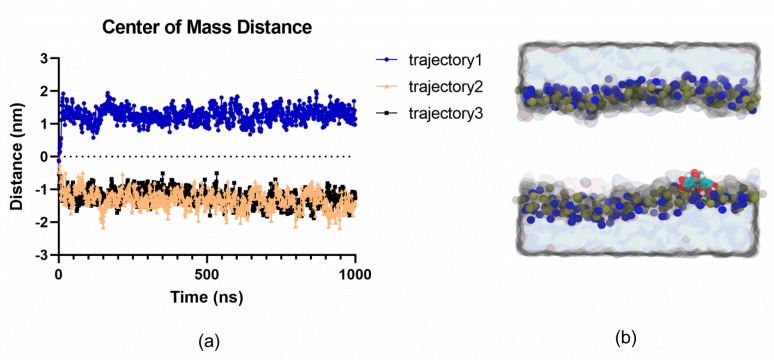
Molecular dynamics simulations show the preferential localization of EGCG in the headgroup region of a POPC membrane. (**a**) Localization of EGCG molecules along 1-microsecond simulation runs with respect to the bilayer center (0 nm). (**b**) Representative snapshot highlighting the preferential localization of the EGCG in the headgroup region of a POPC bilayer (phosphate and nitrogen atoms are shown as blue and brown spheres, respectively, the solution is shown as a grey surface and lipid tails are omitted for clarity. The EGCG molecule is shown as cyan, red, and white spheres).

To obtain a first insight into the mechanism through which EGCG may exert its effect on Na_v_1.5, the interaction between EGCG and a model lipid membrane that mimics the environment in which Na_v_1.5 is found, was characterized. To this effect, molecular dynamics (MD) simulations of a system containing EGCG initially positioned at the center of a 1-palmitoyl-2-oleoyl-sn-glycero-3-phosphocholine (POPC) bilayer, were carried out. Molecular dynamics simulations enable us to investigate the time-dependent dynamical and structural properties of systems at the atomistic level. Three 1 µs simulations suggested that EGCG did not preferentially stay in the center of the bilayer, but that it spontaneously and consistently relocated to the membrane headgroup region (Figure 5). Over the course of the simulations, no signs of spontaneous crossing from one leaflet to the other were observed, indicative of a relatively large free energy barrier associated with EGCG transport across the membrane, that is not easily crossed on the microsecond timescale. This finding is consistent with the molecule’s amphiphilic nature. These results indicate that EGCG may not spontaneously permeate the membrane and become able to block the Na_v_1.5 channel via the entrance to the internal cavity via the inner pore mouth.

## 3. Discussion

The present study aimed to characterize the biophysical mechanism underlying the EGCG effect on the cardiac sodium channel Na_v_1.5.

Our results showed that EGCG inhibited the I_Na_ current in a concentration-dependent manner either at a holding potential of −100 or at −80 mV. The IC_50_ of EGCG was much lower at a holding potential of −80 mV compared to −100 mV, (IC_50_ = 32.4 ± 3.6 µM and 2.1 ± 1 µM at −100 mV and −80 mV, respectively). This demonstrates that the membrane holding potential affects the affinity of EGCG to Na_v_1.5, suggesting that EGCG interacts with a higher affinity to the inactivated state of the cardiac sodium channel. Similar results have been reported by Kang et al., showing a dose and state-dependent inhibition of I_Na_ current in the presence of EGCG [30]. However, the obtained IC_50_ were different from those published by these authors (100 µM and 24.6 µM at holding potentials of −90 mV and −70 mV, respectively [30]). These discrepancies are likely related to different experimental conditions used in the two studies. Kang et al., have stably transfected Na_v_1.5 in HEK293 cells in the absence of its regulatory subunit Na_v_-β1, investigated the pharmacological effect of ECGC at 35 °C, and used −90 or −70 mV as holding potentials to generate I_Na_ currents. In contrast to these conditions, we have transiently transfected tsA-201 cells with Na_v_1.5 in the presence of Na_v_-β1, recorded I_Na_ currents at room temperature (23–25 °C), and used −100 or −80 mV as holding potentials to generate I_Na_ currents. These protocol changes may induce noticeable differences. Indeed, since Na_v_-βs are strong modulators of Na_v_1.5 electrophysiological properties [14], the sensitivity of the cardiac sodium channel towards EGCG may be affected by the presence or absence of Na_v_-β1. Furthermore, conducting experiments at elevated temperatures may induce the degradation of EGCG and its epimerization to gallocatechin-3-gallate (GCG) [31], which could trigger different biophysical modifications of I_Na_ current as it was shown in rat dorsal root ganglion neurons [32]. On the other hand, under the same conditions, the activity of EGCG on Na_v_1.5 could be altered as it was described for other I_Na_ inhibitors such as flecainide, ajmaline, and ranolazine [33,34].

The biophysical mechanism of Na_v_1.5 inhibition by EGCG was further investigated by characterizing the biophysical gating properties of these channels in the presence of 30 µM EGCG. Under these conditions, EGCG shifts the voltage-dependence of inactivation curve toward more negative potentials by 7.6 mV, slows the inactivation kinetics, and delays the recovery from fast inactivation, indicating that EGCG binds to the inactivated state of Na_v_1.5. However, EGCG had no effect on the voltage-dependence of activation and significantly affected the activation curve slope. The modification of the activation slope may be caused by a more efficient clamp of the membrane potential in the presence of 30 µM EGCG. Indeed, even with optimal compensation for series resistance (Rs), the remaining uncompensated Rs combined with large and rapid I_Na_ currents let a voltage error that could produce a loss of the voltage clamp. Thus, a reduced sodium current magnitude, as it has been noticed in the presence of EGCG, could decrease the resulting series resistance error and improve the voltage clamp quality.

On the other hand, the observed shift of the steady-state of inactivation is consistent with what has been previously described for Na_v_1.5 [30]. One should note that the EGCG effects on the other biophysical properties were mainly investigated on the neuronal Na_v_ channels [32,35,36]. These published results are in concordance with the EGCG effect on the fast inactivation kinetics, and recovery from inactivation of the cardiac sodium channels.

With regards to the activation process, Kim et al. observed that 1µM EGCG did not affect the voltage-dependence of activation of tetrodotoxin-sensitive and resistant I_Na_ currents in rat dorsal root ganglion neurons [36], which was consistent with the results of the present study studying the cardiac sodium current. In contrast, Deng et al. showed a negative shift of the activation curve in the presence of EGCG in primary cultures of rat hippocampal neurons [35]. From our perspective, these discrepancies could be related to the quality of the membrane potential clamp. Indeed, based on the sodium current traces presented in Deng’s study, a sub-optimal voltage-clamp quality of these currents is observed, which makes any interpretation difficult [35].

Furthermore, our investigations suggested that there is little or no use-dependent block of EGCG on Na_v_1.5. The absence of the UDB could be explained by the slight modification of the recovery from fast inactivation. Indeed, in the presence of EGCG, the almost “normal” recovery from fast inactivation prevents an additional progressive accumulation of inactivated Na_v_1.5, which represents the preferential binding state of EGCG. These findings are consistent with those obtained in other studies testing the effect of gallocatechin-3-gallate on the tetrodotoxin-resistant voltage-gated sodium channels in rat dorsal root ganglion neurons [32].

In a first attempt to characterize the molecular mechanism through which EGCG may exert its effect on Na_v_1.5, we investigated how EGCG interacts with the lipidic environment surrounding Na_v_1.5. Indeed, relatively hydrophobic compounds such as EGCG have been hypothesized to act on membrane proteins either via direct interaction, via the effect on the membrane, or both. Voltage-gated sodium channels, in particular, have fenestrations lining their pore domain, which enable modulators to access the pore via the membrane. We thus sought to gain a first insight into how Na_v_ channels may be modulated by EGCG by characterizing where this compound localized preferentially in a membrane. This was done by carrying out MD simulations, which provided a detailed atomistic view of the behavior of EGCG when it was placed in a model lipidic membrane: Calculations were initiated with EGCG located at the center of the POPC bilayer. The obtained results suggest that EGCG is spontaneously relocated to the membrane headgroup region.

The predicted re-localization of EGCG to the membrane head group is consistent with the results of Salazar et al., which have demonstrated that EGCG modifies the activity of a membrane-bound protein upon binding to the phospholipid head groups of the red blood cell membranes [37]. Similarly, Ingólfsson et al. have shown that the biological effects of green tea catechins could be attributed to multiple molecular interactions because the catechins accumulate at the bilayer/solution interface and modify bilayer properties that can influence gramicidin channel function [38]. However, MD simulations results cannot exclude the possibility that EGCG exerts its biophysical effect via access to its binding site, on Na_v_1.5, through the cell membrane. Indeed, as shown for local anesthetics and antiarrhythmic drugs [39], the EGCG blocking effect could also be controlled by the channel’s fenestrations.

The current findings support the hypothesis that EGCG could be used as an anti-arrhythmic drug in the setting of Na_v_1.5 gain-of-function mutations; as has been suggested by Boukhabza et al. These authors have predicted that EGCG may limit the cardiac cells hyperexcitability related to the presence of Na_v_1.5-R222Q and Na_v_1.5-I141V mutants [20]. On the other hand, the present study demonstrated that the membrane holding potential affects the affinity of EGCG to Na_v_1.5, suggesting that EGCG interacts with a higher affinity to the inactivated state of the cardiac sodium channel. Thus, we could expect an atrial-selective block of Na_v_1.5 channels due to a more depolarized resting membrane potential in the atria compared to the ventricles. Then, a higher beneficial effect of EGCG could be observed in the setting of atrial hyperexcitability phenotypes.

Nonetheless, it remains somehow challenging to interpret the clinical relevance of our results even in the context of cardiac hyperexcitability phenotypes. Many in vitro studies have shown that the effective concentrations of EGCG to target relevant pathophysiological pathways are typically ranged from 1 to 1000 µmol/L. However, the pharmacological disposition of EGCG in humans indicates that its serum concentration is in the nanomolar scale [40,41,42,43,44,45,46,47,48]. In addition, EGCG is well known for its chemical instability under physiological conditions due to its easy degradation and metabolism [31,49,50]. Therefore, any potential impact of EGCG intake may reflect the combined effects of EGCG and its metabolites.

In conclusion, the present study suggests that EGCG may modulate the biophysical properties of Na_v_1.5 via access to its binding site through the cell membrane or via a bilayer-mediated mechanism. These results constitute a first step towards the characterization of the biophysical mechanism underlying the inhibitory action of EGCG on Na_v_1.5. However, the exact molecular interactions underlying these effects are still unknown and remain to be studied.

## 4. Materials and Methods

### 4.1. Cell Culture and Transfection

TsA-201 cells were cultured at 37 °C in Dulbecco’s Modified Eagle Medium (DMEM) supplemented with 10% Fetal Bovine Serum (FBS), 4 mM glutamine, and a cocktail of streptomycin-penicillin antibiotics in a humidified atmosphere of 5% CO_2_ and 95% air. All cell medium components, except glutamine (Sigma-Aldrich), were purchased from Gibco (Fisher Scientific, Zurich, Switzerland).

For electrophysiological experiments, tsA-201 cells were transiently transfected with DNA complexed to JetPEI (Polyplus-transfection) according to the manufacturer’s instructions. DNA concentrations were 1 µg of pCDN3.1-Na_v_1.5 and 1 µg of pIRES-hβ1-CD8. The resulting Na_v_1.5 protein was a splice variant lacking glutamine at position 1077 (Q1077del). Eight hours after transfection, the cells were isolated and seeded in plastic Petri dishes at low density.

### 4.2. Cellular Electrophysiology

Twenty-four hours after transfection, the resulting sodium current (I_Na_) was recorded at room temperature (23–25 °C), under whole-cell voltage-clamp conditions with Axon PClamp 10 software through an A/D converter (Digidata 1440A) using an Axopatch 200B (all Axon Instruments Molecular Devices Corp., CA, USA). Series resistances were compensated (up to 90% compensation), and the residual capacitive currents were canceled using a P/4 protocol. The cells were bathed with an extracellular solution containing (in mmol/L): NaCl 50, NMDG-Cl 80, CsCl 5, MgCl_2_ 1.2, CaCl_2_ 2, HEPES 10, glucose 5. The pH was adjusted to 7.4 with CsOH. Glass pipettes (tip resistance: 1.5 to 3 MΩ) were filled with an intracellular medium containing (in mmol/L): Na_2_ATP 5, CsAsp 70, CsCl 60, EGTA 11, CaCl_2_ 1, MgCl_2_ 1, HEPES 10. pH was adjusted to 7.2 with CsOH.

Activation properties of Na_v_1.5 channels were determined from I/V relationships by normalizing the sodium current peak to the driving force. Steady-state inactivation was measured with a standard double-pulse protocol. Cells were held at −100 mV before the application of 500 ms conditioning pulses, ranging from −130 to −20 mV. Then, a 20 ms test pulse to −20 mV was applied. The measured current was then normalized to the maximum peak current recorded after conditioning pulses (I/Imax) and plotted as a function of conditioning step voltage. Activation and inactivation curves were fit to a Boltzmann equation.

For the pharmacological investigation, the peak current amplitudes at different EGCG concentrations were subtracted from the current obtained with the control solution and were normalized to the control value:

Inhibition Percentage (%) = [(Icontrol − IEGCG)/Icontrol] × 100

IC_50_ was calculated according to Quest GraphTM IC_50_ Calculator (Quest GraphTM IC50 Calculator). AAT Bioquest, Inc., 24 Apr. 2019, https://www.aatbio.com/tools/ic50-calculator).

### 4.3. Chemicals

Epigallocatechin-3-Gallate (EGCG, Sigma-Aldrich, CAS Number 989-51-5) was dissolved directly into the external solutions. Fresh EGCG solutions were prepared before each experiment. During the same day, EGCG solutions were renewed every 5 h to avoid compound degradation (e.g., brown discoloration of solutions).

### 4.4. Molecular Dynamics Simulations

The 3D structure of the EGCG molecule was obtained from PubChem Database ((−)-Epigallocatechin gallate, CID = 65064). The system containing an EGCG molecule placed at the center of a 1-palmitoyl-2-oleoyl-sn-glycero-3-phosphocholine membrane and plunged in a 0.1 M NaCl solution was prepared using the CHARMM-GUI server [51,52,53]. The lipid and ions interactions were described using the CHARMM36 (Chemistry at Harvard Macromolecular Mechanics) force field [54], whereas water molecules were modeled using the TIP3P model [55]. Simulations were performed using the NPT ensemble, where the temperature and the pressure were maintained at 310 K and 1 atm, respectively, using Parinello–Rahaman pressure coupling [56] and Nose-Hoover temperature coupling [57]. The cut-off value used to compute non-bonded interactions was set to 12 Å and long-range electrostatic interactions were computed using the particle mesh Ewald method [58]. Three replicas 1 µs simulations were run using the GROMACS package version 2018.4 on our local high-performance computing cluster [59]. Distances between the COM (Center of Mass) of EGCG and the membrane were calculated by using the gmx distance analysis tool of GROMACS. EGCG was parameterized using the SWISS-PARAM software [60].

### 4.5. Data Analysis and Statistical Methods

Currents were analyzed with Clampfit software (Axon Instruments, San Jose, CA, USA). Data were analyzed using a combination of pClamp10, Excel (Microsoft), Igor Pro (WaveMetrics), and Prism (graphpad).

Comparisons between groups were performed with a two-tailed paired Student’s t-test. Data were expressed as mean ± SEM. A *p*-value < 0.05 was considered significant.

## 5. Conclusions

In the current study, we have characterized the effect of epigallocatechin-3-gallate on the cardiac sodium channel. This compound shows a higher affinity for the inactivated state of Na_v_1.5 without any effect on its activation process. In addition, molecular dynamics simulations have predicted the re-localization of EGCG to the membrane headgroup region, suggesting that EGCG may not spontaneously permeate the cell membrane and may exert it effects on Na_v_1.5 by a bilayer mediated mechanism.

## Figures and Tables

**Table 1:** Effect of EGCG on the gating properties of Na_v_1.5 channels.

	Control	30 µM EGCG
Peak current densities at −20 mV (pA/pF)	−146.2 ± 17.8; *n* = 12	−64.3 ± 7.3 ***; *n* = 12
V_1/2_ activation (mV)	−27.4 ± 0.8; *n* = 12	−27.4 ± 1; *n* = 12
Activation slope (mV)	6.7 ± 0.2; *n* = 12	8 ± 2 ***; *n* = 12
Time to peak at −20 mV (ms)	1.12 ± 0.02; *n* = 12	1.16 ± 0.03; *n* = 12
V_1/2_ inactivation (mV)	−79.4 ± 1.3; *n* = 13	−87 ± 0.9 ***; *n* = 13
Inactivation slope (mV)	5 ± 0.2; *n* = 13	5.5 ± 0.2 *; *n* = 13
t1/2 inactivation at −20 mV (ms)	0.43 ± 0.02; *n* = 12	0.56 ± 0.03 **; *n* = 12
Recovery from fast inactivation (ms)		
τfast,1	5.1 ± 0.9; *n* = 5	24.3 ± 7.1 *; *n* = 5
τfast,2	110 ± 28.6; *n* = 5	233.7 ± 37.6 *; *n* = 5

* *p* < 0.05, ** *p* < 0.01, *** *p* < 0.001.

**Table 2:** Characterization of the use-dependent block of EGCG.

	UDB-1 Hz	UDB-10 Hz
EGCG (30 µM)	0.93 ± 0.01 **, *n* = 5	0.90 ± 0.02 *, *n* = 5
Mexiletine (100 µM)	0.86 ± 0.01 ***, *n* = 4	0.21 ± 0.04 ***, *n* = 4

* *p* < 0.05, ** *p* < 0.01, *** *p* < 0.001 versus control.

## Supplementary Materials

The following are available online, Figure S1: EGCG dose-dependent effect on the fast inactivation kinetics. (a) Representative traces of normalized I_Na_ current in the presence of various EGCG concentrations. (b) EGCG effect on I_Na_ inactivation kinetics.
