# Supplementary material for: Biophysical Characterization of Epigallocatechin-3-Gallate Effect on the Cardiac Sodium Channel Nav1.5
Source: Molecules. 2020 Feb 18;25(4):902. doi: 10.3390/molecules25040902 (PMC7070937; doi:10.3390/molecules25040902)
Supplement: Supplementary file 1 [file molecules-25-00902-s001.pdf]

Article

Biophysical Characterization of Epigallocatechin-3-Gallate Effect on the Cardiac Sodium Channel $Na_V1.5$

Mohamed-Yassine Amarouch [1,*], Han Kurt [2], Lucie Delemotte [2] and Hugues Abriel [3,*]

[1] R.N.E Laboratory, Multidisciplinary Faculty of Taza, University Sidi Mohamed Ben Abdellah of Fez, Fez 30000, Morocco
[2] Science for Life Laboratory, Department of Applied Physics, KTH Royal Institute of Technology, Solna, SE-100 44, Sweden; han.kurt@outlook.com (H.K.); lucie.delemotte@scilifelab.se (L.D.)
[3] Institute of Biochemistry and Molecular Medicine (IBMM), University of Bern, 3012 Bern, Switzerland
* Correspondence: mohamed.amarouch@usmba.ac.ma (M.-Y.A.); hugues.abriel@ibmm.unibe.ch (H.A.)

Academic Editors: Saverio Bettuzzi and Jean-Marc Sabatier

Received: 26 December 2019; Accepted: 15 February 2020; Published: date

Supplementary Figure

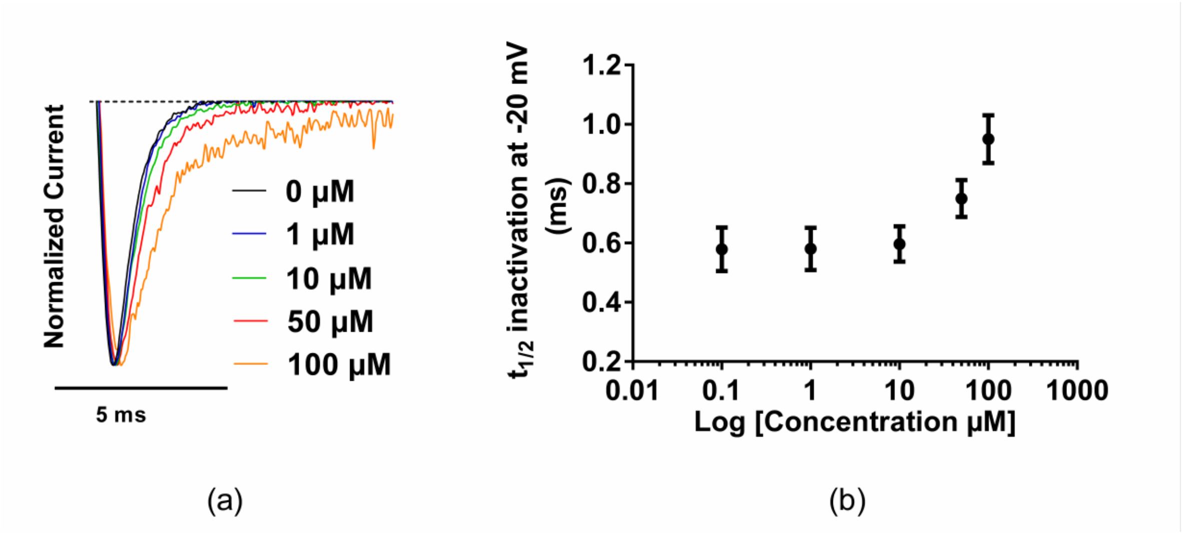

Supplementary Figure S1: EGCG dose-dependent effect on the fast inactivation kinetics. (a) Representative traces of normalized I_{Na} current in the presence of various EGCG concentrations. (b) EGCG effect on I_{Na} inactivation kinetics.
